# Reducing Sintering Temperature While Optimizing Electrical Properties of BCZT-Based Lead-Free Ceramics by Adding MnO_2_ as Sintering Aid

**DOI:** 10.3390/ma18081888

**Published:** 2025-04-21

**Authors:** Xinlin Yang, Bijun Fang, Shuai Zhang, Xiaolong Lu, Jianning Ding

**Affiliations:** 1 School of Materials Science and Engineering, Jiangsu Collaborative Innovation Center of Photovoltaic Science and Engineering, State Key Laboratory of Photovoltaic Science and Technology, National Experimental Demonstration Center for Materials Science and Engineering, Changzhou University, Changzhou 213164, China; s23010805022@smail.cczu.edu.cn (X.Y.); shuaizhang@cczu.edu.cn (S.Z.); xllu@cczu.edu.cn (X.L.); 2 School of Mechanical Engineering, Yangzhou University, Yangzhou 225127, China

**Keywords:** BCZT-based ceramics, sintering aid MnO_2_, sintering temperature, electrical performance

## Abstract

In order to reduce the sintering temperature, MnO_2_ was used as a sintering aid to prepare [(Ba_0.85_Ca_0.15_)_0.999_(Dy_0.5_Tb_0.5_)_0.001_](Zr_0.1_Ti_0.9_)O_3_-x mol% MnO_2_ (BCDTZT-x mol% MnO_2_, x = 0.05, 0.2, 0.4, 0.6, 0.8, 1, 1.5, 3) lead-free piezoelectric ceramics in which the effects of the MnO_2_ doping amount and sintering temperature on the phase structure, sintering behavior, and electrical properties of the BCDTZT-x mol% MnO_2_ ceramics were systematically analyzed. All ceramics have a single perovskite structure and coexist in multiple phases. The optimal sintering temperature was reduced from 1515 °C to 1425 °C, and the density of all ceramics was increased as compared with the undoped ceramic, reaching a maximum of 5.38 g/cm^3^ at x = 0.8 mol%. An appropriate MnO_2_ doping amount of 0.4 mol% could effectively suppress oxygen vacancies and improve electrical properties, resulting in the best comprehensive performance of the ceramics, with a dielectric constant maximum of 12,817, a high piezoelectric constant of 330 pC/N, and good strain value (S_max_ = 0.118%) and low strain hysteresis (Hys = 2.66%). The calculation of activation energy indicated that the high-temperature conductivity was dominated by oxygen vacancies in all ceramics. The results showed that the appropriate introduction of MnO_2_ as a sintering aid could improve the performance of BCZT-based ceramics while reducing the sintering temperature, presenting high practical application value in the fields of low electric field sensors and actuators.

## 1. Introduction

With the rise of lead-free piezoelectric materials, the development and preparation of lead-free piezoelectric ceramics with high performance and low energy consumption have become important research hotspots [1,2,3,4,5,6]. Liu and Ren prepared (Ba_0.85_Ca_0.15_)(Zr_0.1_Ti_0.9_)O_3_ (BCZT) lead-free ceramics in 2009 that had piezoelectric properties comparable to the Pb(Zr, Ti)O_3_ (PZT)-based piezoelectric ceramics [7,8]. This discovery sparked a global upsurge of research on lead-free ceramics.

Subsequent studies have shown that BCZT ceramics are some of the most likely materials to replace lead-based piezoelectric ceramics in the future [2,3,5,6,8,9]. Although BCZT ceramics have excellent electrical properties, they still have significant disadvantages. As is well known, a high sintering temperature is an unfavorable condition for the preparation of BCZT ceramics [7]. Compared with lead-based ceramics with a sintering temperature of around 1250 °C, the sintering temperature of BCZT ceramics can reach as high as 1475 °C [7]. Excessive high sintering temperatures will result in significant energy consumption and higher production cost. Currently, research on reducing the sintering temperature of BCZT ceramics mainly involves changing the preparation process and adding sintering aids [10,11]. Although changing the preparation process can greatly reduce the sintering temperature of ceramics, their electrical properties will also deteriorate accordingly. Adding sintering aids can lower the sintering temperature while maintaining the original properties of ceramics, making them more practically applicable [11,12,13,14,15].

Many studies have shown that MnO_2_ doping can reduce oxygen vacancies, leading to improve the electrical resistivity, reduce dielectric loss, and enhance the piezoelectric properties of piezoceramics [16,17,18,19,20,21,22]. Peng et al. systematically studied the effects of MnO_2_ doping and sintering atmosphere on the properties of 0.05%Nb_2_O_5_-BaTiO_3_ ceramics. X-ray photoelectron spectroscopy (XPS) analysis showed that MnO_2_ doping could effectively suppress oxygen vacancies, increase resistivity, and reduce dielectric loss [19]. In order to improve ferroelectric properties of the NaNbO_3_ ceramics, Li et al. found that adding 1 wt% MnO_2_ as a sintering aid significantly increased the dielectric constant and obtained a strong ferroelectricity with a maximum polarization intensity of 84.6 μC/cm^2^ at 60 kV/cm, providing a simple way to improve the ferroelectric properties of ceramics [20]. Lin et al. prepared 0.62BiFeO_3_-0.23PbTiO_3_-0.15BaTiO_3_-x mol% MnO_2_ (BF-PT-BTMn) ceramics. When the MnO_2_ content was 0.8 mol%, the dielectric loss of BF-PT-BTMn ceramics decreased from 0.05 to 0.008 while the piezoelectric constant d_33_ increased from 163 pC/N to 238p C/N. At the same time, the Curie temperature (T_C_) remained almost unchanged, and the ceramics exhibited good piezoelectric thermal stability [21]. Mulualem et al. doped different contents of MnO_2_ into BCZT ceramics, and the sintering temperature was reduced from 1500 °C to 1300 °C. When the MnO_2_ doping amount was 0.4 mol%, the sample’s theoretical density reached 96.5%, and excellent piezoelectricity (d_33_~534 pC/N) was obtained [22].

In this work, Dy/Tb co-doped [(Ba_0.85_Ca_0.15_)_0.999_(Dy_0.5_Tb_0.5_)_0.001_](Zr_0.1_Ti_0.9_)O_3_ ceramics were used as a studied matrix based on our previous experiments, in which the adverse effect caused by excessive single-rare-earth-element doping could be suppressed through the co-doping of dual rare earth elements. Meanwhile, Dy^3+^ and Tb^3+^ are adjacent rare earth elements with similar chemical properties in the periodic table, and they exhibit dipole–dipole interactions with energy transitions reported in the Tb^3+^/Dy^3+^ doped Bi_2_Ti_2_O_7_ glass ceramics and Dy^3+^/Tb^3+^ co-doped K_3_YF_6_ transparent oxyfluoride glass ceramics [23,24]. However, the sintering temperature of [(Ba_0.85_Ca_0.15_)_0.999_(Dy_0.5_Tb_0.5_)_0.001_](Zr_0.1_Ti_0.9_)O_3_ ceramic was too high (1515 °C) and its density of 4.99 g/cm^3^ could be increased by introducing MnO_2_ as a sintering aid. Firstly, the optimum sintering temperature was determined using the [(Ba_0.85_Ca_0.15_)_0.999_(Dy_0.5_Tb_0.5_)_0.001_](Zr_0.1_Ti_0.9_)O_3_-0.4 mol% MnO_2_ composition. Then, the effects of the MnO_2_ doping amount prepared at a sintering temperature of 1425 °C were further investigated. Compared with the undoped [(Ba_0.85_Ca_0.15_)_0.999_(Dy_0.5_Tb_0.5_)_0.001_](Zr_0.1_Ti_0.9_)O_3_ sample, the optimum sintering temperature was decreased from 1515 °C to 1425 °C, and the sample with a 0.4 mol% MnO_2_ doping amount achieved improved dielectric (maximum dielectric constant ε_max_ = 12,817) and piezoelectric properties (d_33_ = 330 pC/N, maximum electro-induced strain S_max_ = 0.118%), presenting promising application prospects in preparing BCZT-based ceramics.

## 2. Experimental Procedure

[(Ba_0.85_Ca_0.15_)_0.999_(Dy_0.5_Tb_0.5_)_0.001_](Zr_0.1_Ti_0.9_)O_3_-x mol% MnO_2_ (BCDTZT-x mol% MnO_2_, x = 0.05, 0.2, 0.4, 0.6, 0.8, 1, 1.5, 3) lead-free piezoelectric ceramics were prepared by solid-state reaction method. The raw materials were barium carbonate (BaCO_3_, 99%), calcium carbonate (CaCO_3_, 99%), dysprosium oxide (Dy_2_O_3_, 99.9%), terbium oxide (Tb_4_O_7_, 99.5%), zirconium dioxide (ZrO_2_, 99%), titanium dioxide (TiO_2_, 99.99%), and manganese dioxide (MnO_2_, 99.95%). All raw materials for preparing BCDTZT were fully dried in an oven at 120 °C for 6 h and weighed according to the required stoichiometric ratio and mixed by ball milling. Then, the well-mixed powder was calcined in a muffle furnace at 1200 °C for 4 h. MnO_2_ was used as a sintering aid and added into the calcined powder with different doping amounts and mixed thoroughly. After granulation with addition of 8 wt% polyvinyl alcohol aqueous solution as a binder, disk shape pellets were pressed with a diameter of 12 mm and a thickness of 1 mm using an alloy-steel mold. The green pellets were removed the binder by holding at 550 °C for 2 h separately and then were sintered at temperatures ranging from 1350 °C to 1450 °C for 3 h using pure ZrO_2_ as a covering powder.

The upper and lower surfaces of the sintered ceramics were polished to obtain parallel and flat surfaces and silver electrode was formed by manual-printed silver paste and heat-treated at 650 °C for 0.5 h for electrical performance characterization. The crystalline structure of BCDTZT-x mol% MnO_2_ ceramics was analyzed by Rigaku D/max-2500/PC X-ray diffractometer (XRD) (Tokyo, Japan) using polished samples. The microstructure of ceramic free surfaces was observed using JSM-IT100 scanning electron microscope (SEM) (JEOL, Tokyo, Japan). The dielectric properties–temperature–frequency relationship and complex impedance spectra of samples were measured with Parulab HDMS-1000 system (Wuhan Partulab Technology Co., Ltd., Wuhan, China). The hysteresis loop (P-E) and strain curve (S-E) were measured at room temperature using Radiant Precision Premier LC II (Radiant Technologies Inc., Albuquerque, NM, USA) under 25 kV/cm at 1 Hz. The MPD PLUS polarizing device was used to pole ceramics under direct current poling (DCP) method for 3 min under 25 kV/cm at room temperature. The d_33_ of samples was measured using ZJ-6A meter (Institute of Acoustics, Chinese Academy of Sciences, Beijing, China). The electromechanical coupling coefficient K_p_ and mechanical quality factor Q_m_ were characterized using TH2826 LCR (Changzhou Tonghui Electronic Co., Ltd., Changzhou, China) by resonance–resonance technique.

## 3. Results and Discussion

This work first prepared the BCDTZT-0.4 mol% MnO_2_ ceramics and investigated the influence of the sintering temperature. Based on the optimized sintering temperature searched, the influence of MnO_2_ doping amount on the structure and performance of BCDTZT ceramics was further studied.

**Figure 1:**
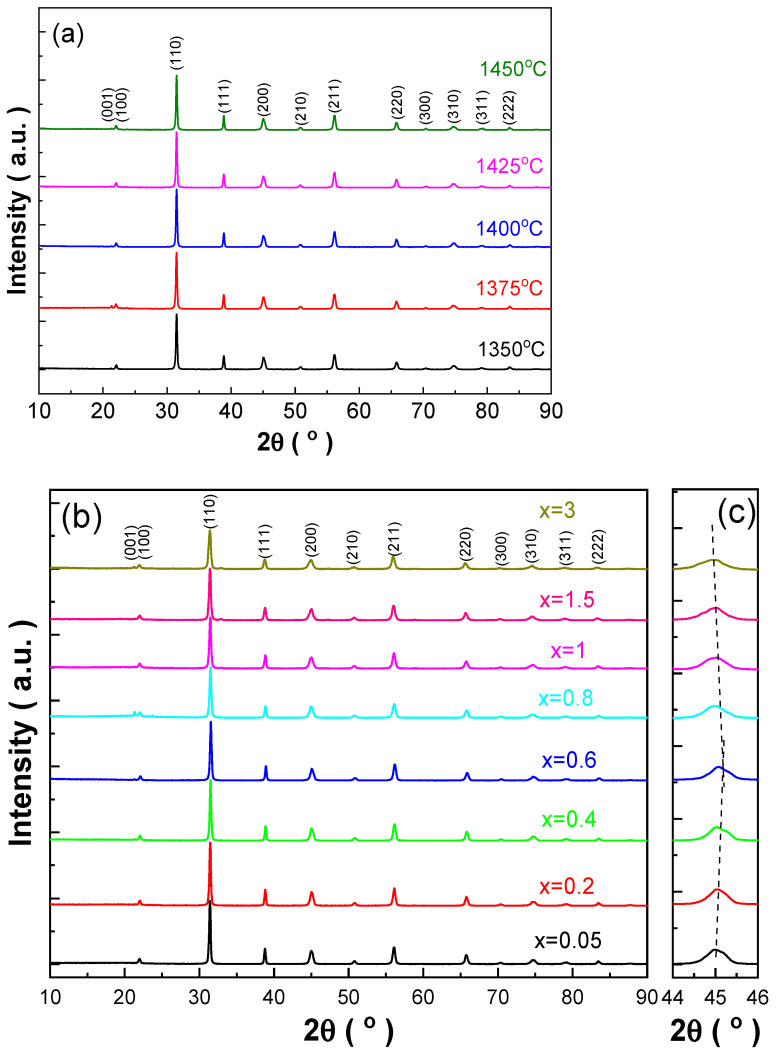
(**a**) XRD patterns of BCDTZT-0.4 mol% MnO_2_ ceramics prepared at different sintering temperatures; (**b**) XRD patterns of BCDTZT-x mol% MnO_2_ ceramics prepared at 1425 °C; (**c**) amplified XRD patterns around (200) peak of BCDTZT-x mol% MnO_2_ ceramics.

Figure 1a,b show the XRD patterns of BCDTZT-0.4 mol% MnO_2_ ceramics sintered at different temperatures and BCDTZT-x mol% MnO_2_ (x = 0.05, 0.2, 0.4, 0.6, 0.8, 1, 1.5, 3) ceramics prepared by sintering at 1425 °C, respectively. The XRD peak position results indicate that all samples exhibited a pure perovskite structure and no secondary impurity phase was detected. These results prove that the introduction of MnO_2_ does not alter the perovskite crystal structure and completely entered the lattice of BCDTZT ceramics, forming a solid solution. All BCDTZT-x mol% MnO_2_ ceramics present a typical perovskite structure similar to that of the standard material Ba_0.91_Ca_0.09_Zr_0.05_Ti_0.95_O_3_ with JCPDS PDF # 00-056-1033, based on which all diffraction peaks were indexed, including weak diffraction reflection {100}.

Figure 1c shows an enlarged local view of the (200) diffraction peak of BCDTZT-x mol% MnO_2_ ceramics. The position of the (200) diffraction peak first shifted towards a higher 2θ angle and then towards a lower 2θ angle with the increase in the MnO_2_ doping amount. According to Bragg’s law,

(1) $$n\lambda =2d_{hkl}\sin\theta .$$

When the 2θ angle value of XRD diffraction peaks increases, the interplanar spacing decreases and the lattice volume shrinks [25]. There are two different valence states of Mn^2+^ and Mn^4+^ in the Mn ion introduced by MnO_2_ doping. When x < 0.6, the lattice volume shrank due to the six-coordinated number (CN) Mn^4+^ (r = 0.53 Å) occupying Zr^4+^ (r = 0.72 Å) and Ti^4+^ (r = 0.605 Å) at the B-site. When the MnO_2_ doping content increased to 3 mol%, 6-CN Mn^2+^ (r = 0.83 Å) with a larger ionic radius entered a dominance state, which led to an increase in the lattice volume, and the 2θ value moved to a lower angle. Therefore, the introduction of MnO_2_ into BCDTZT ceramics to form a solid solution will lead to lattice distortion and electrovalence mismatch, which is conducive to ion diffusion and thus reduces the sintering temperature of preparing BCZT-based ceramics [17,22].

**Figure 2:**
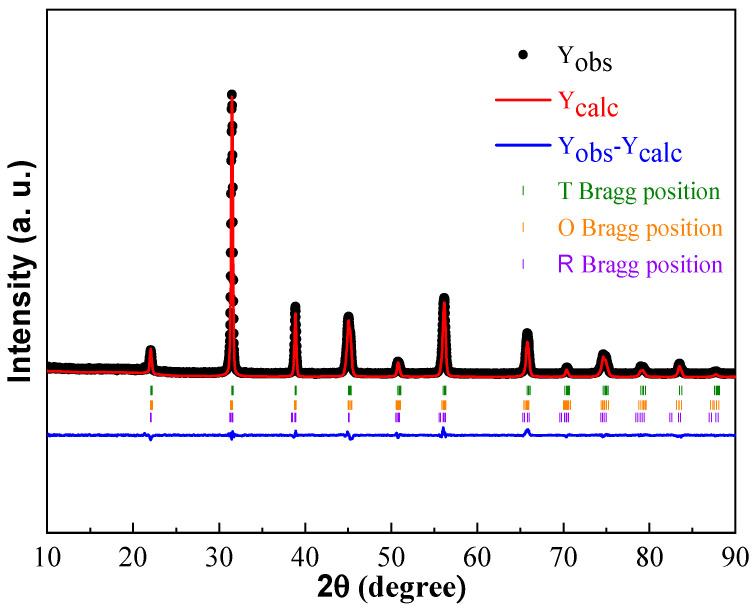
XRD Rietveld refinement of BCDTZT-0.4 mol% MnO_2_ ceramic sintered at 1425 °C.

When the MnO_2_ doping amount was less than 0.8 mol%, significant asymmetry and a broadening of the (200) diffraction peak were observed, indicating the existence of a tetragonal phase in the ceramics [22,26,27]. As the content of MnO_2_ further increased, the splitting gradually disappeared and the content of tetragonal phase decreased, indicating the existence of composition-induced phase transition [22,26]. To further explain the existence of phase transition, the XRD patterns of BCDTZT- x mol% MnO_2_ ceramics were refined using the GSAS Rietveld technique, and the refinement results are shown in Figure S1 and Figure 2 and Table 1. From Table 1, it can be seen that all samples had R_wp_ < 10% and χ^2^ < 4.7, indicating that the refined results were within an acceptable range and had high credibility. Through the phase structure analysis of the samples, it was found that there were three phases coexisting in the range of x = 0.05 to x = 0.8, namely the orthogonal phase (O, Amm2), rhombohedral phase (R, R3m), and tetragonal phase (T, P4mm), proving that the ceramics were located at the morphotropic phase boundary (MPB) region [26,27,28]. When x was greater than 0.8, a new cubic phase (C, Pm3m) was generated. When the three phases coexisted (x = 0.05–0.8), the content of the tetragonal phase increased first and then decreased with the increase in the MnO_2_ doping amount, reaching the maximum content at x = 0.4. At this composition, the sum of content of the Amm2 and P4mm phases was greater than 85% and the ratio was close to 1, indicating that the ceramic was mainly located at the O-T phase boundary. According to relevant studies, ceramics located at the MPB can be advantageous for obtaining excellent piezoelectric properties, and compared to those located at the R-T phase boundary, the materials around the O-T phase boundary can achieve higher piezoelectric properties due to the easier polarization rotation and greater lattice softening [26,27,29]. The appearance of the cubic phase will cause a decrease in the remnant polarization of ceramics, which will reduce the piezoelectric properties [30].

**Figure 3:**
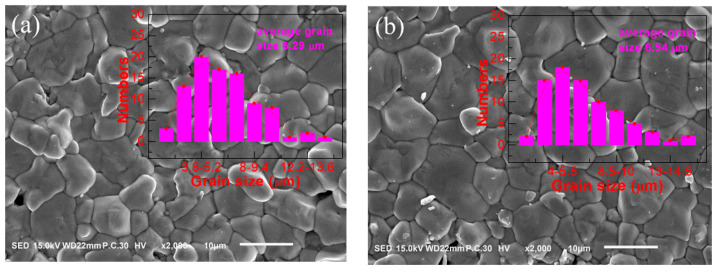

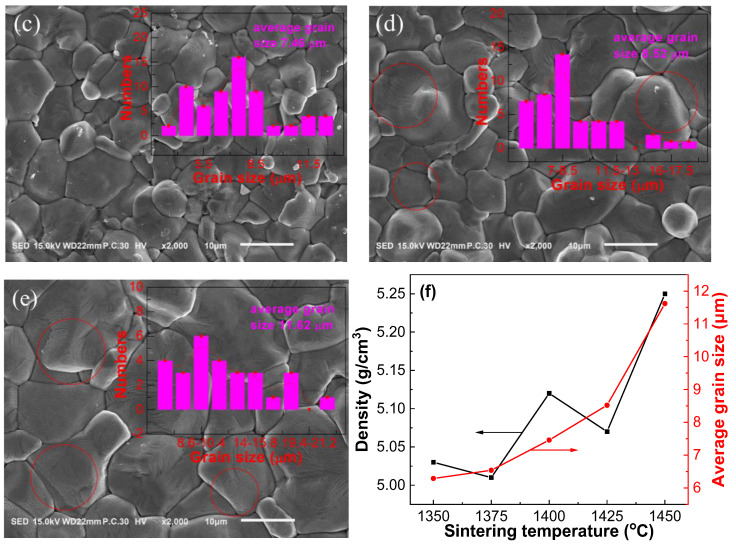
SEM microstructures and grain size distributions of BCDTZT-0.4 mol% MnO_2_ ceramics prepared at different sintering temperatures: (**a**) 1350 °C; (**b**) 1375 °C; (**c**) 1400 °C; (**d**) 1425 °C; (**e**) 1450 °C. (**f**) Changes in density and average grain size of BCDTZT-0.4 mol% MnO_2_ ceramics with sintering temperature.

In order to understand the effects of sintering temperature and the addition amount of MnO_2_ as a sintering aid on the microstructures of BCDTZT ceramics, the samples were amplified 2000 times by an SEM. Figure 3a–e show the microstructure of BCDTZT-0.4 mol% MnO_2_ ceramics sintered at 1350 °C to 1450 °C, and their enlarged SEM images are shown in Figure S2. The samples exhibited adequate grain growth, wherein clear grain boundaries and uniform grain size distribution were obtained. At a sintering temperature of 1350 °C, there were still a few pores in the sample, whereas the pores gradually disappeared as the sintering temperature increased. From Figure 3f, it can be seen that both the ceramic density and average grain size increased with an increase in the sintering temperature. At 1450 °C, the optimal density of 5.25 g/cm^3^ and the maximum average grain size of 11.62 μm were achieved.

**Figure 4:**
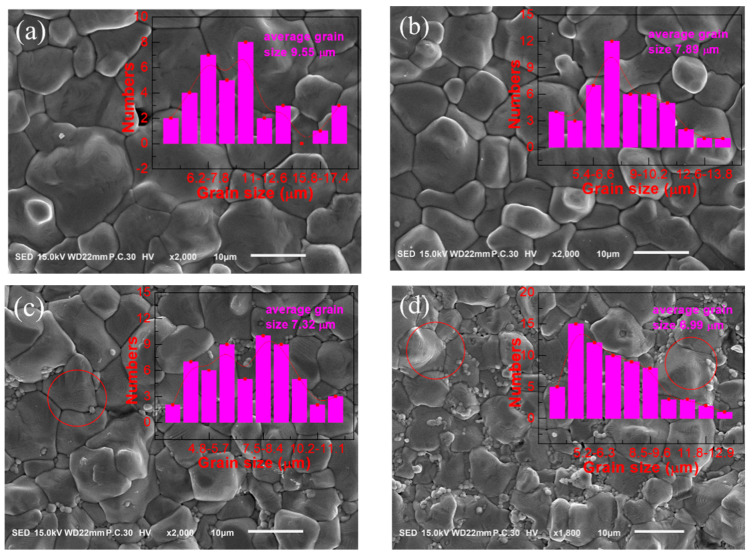

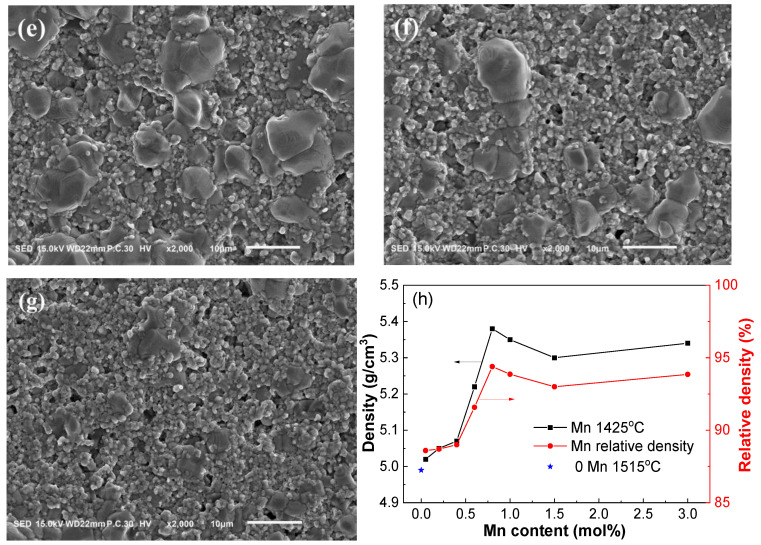
SEM microstructures and grain size distributions of BCDTZT-x mol% MnO_2_ ceramics sintered at 1425 °C: (**a**) x = 0.05; (**b**) x = 0.2; (**c**) x = 0.6; (**d**) x = 0.8; (**e**) x = 1; (**f**) x = 1.5; (**g**) x = 3. (**h**) Density variation in BCDTZT-x mol% MnO_2_ ceramics.

The purpose of this work was using MnO_2_ as a sintering aid to decrease sintering temperature; therefore, 1425 °C was determined as the sintering temperature to investigate the effects of different MnO_2_ doping amounts on BCDTZT ceramics. From Figure 3d and Figure 4, it can be seen that with the increase in the MnO_2_ doping amount, the average grain size first decreased, abnormally increased at 0.4 mol%, and then decreased continuously. According to literature reports, ions with small ionic radii exhibit higher ion mobility during sintering; therefore, the grain size decreased due to the introduction of MnO_2_ in Mn^4+^ valence [17,31]. Equations (2)–(4) represent the defect formation equations of BCDTZT-x mol% MnO_2_ ceramics using the Kröger–Vink symbol [17,22,32].

(2) $${Mn}^{4+}\overset{{Zr}^{4+}{/Ti}^{4+}}{\to }{Mn}_{Zr/Ti}^{\times }$$

(3) $${Mn}^{2+}\overset{{Zr}^{4+}{/Ti}^{4+}}{\to }{Mn}_{Zr/Ti}^{\prime \prime }+V_{O}^{\cdot \cdot }$$

(4) $$O+V_{O}^{\cdot \cdot }+2e^{\prime }\to O_{O}^{\times }$$

When x = 0.4, the increase in grain size may have been due to the presence of Mn^2+^ with a larger ionic radius, which also led to the formation of oxygen vacancy defects, reduced the activation energy of reaction, lowered the potential barrier, and promoted grain growth. When the MnO_2_ doping content was greater than 1 mol%, excessive MnO_2_ introduction would cause the aggregation of manganese at grain boundaries, inhibiting the growth of large grains and forming small grains, also known as the segregation phenomenon [33,34,35]. Meanwhile, excessive higher sintering temperatures could also generate a liquid phase in ceramics, resulting in significant porosity [35]. So, as shown in Figure 4h, with the increase in the MnO_2_ addition content, the bulk density and relative density first increased and then decreased, reaching the maximum value at x = 0.8 (5.38 g/cm^3^). However, compared with the undoped BCDTZT ceramic with density of 4.99 g/cm^3^, the density of all MnO_2_-doped samples still presented a significant improvement prepared at the same sintering temperature of 1425 °C. Compared with the pure BCDTZT ceramic with an average grain size of 17.2 μm, the addition of MnO_2_ reduced the grain size of all MnO_2_-doped BCDTZT ceramics, which may have been due to the generation of lattice strain energy and hindering grain boundary migration caused by introducing ions with different ionic radii [17].

From the circular markings shown in Figure 3, Figure 4 and Figure S2, it can be seen that a stepped ferroelectric domain structure appeared in the large grains, which was believed to be the 90° domain wall of the T phase. As the MnO_2_ doping amount increased, the stepped domain wall gradually disappeared, which also indicated a decrease in the T phase content, and was consistent with the XRD refinement results. Based on the SEM results, it can be seen that the appropriate introduction of low-melting-point MnO_2_ as a sintering aid into the BCDTZT ceramics could effectively improve the density of ceramics while reducing the sintering temperature, which helped enhance the electrical properties of the BCZT-based ceramics.

**Figure 5:**
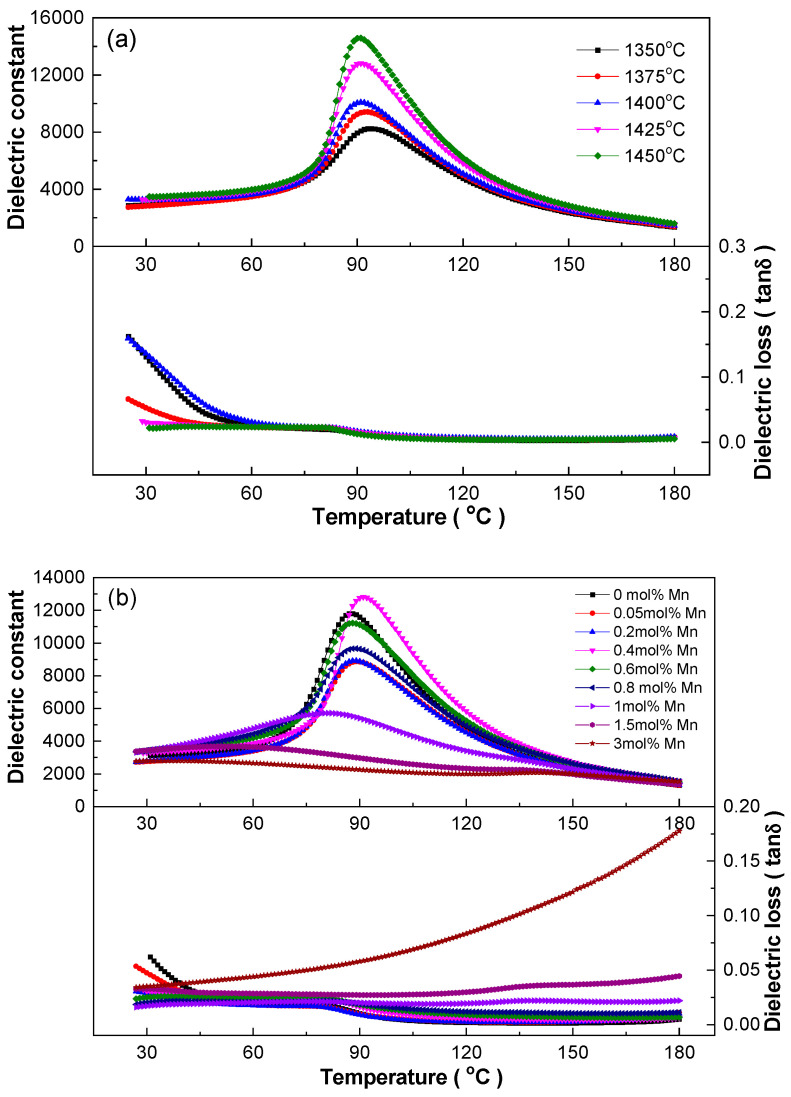

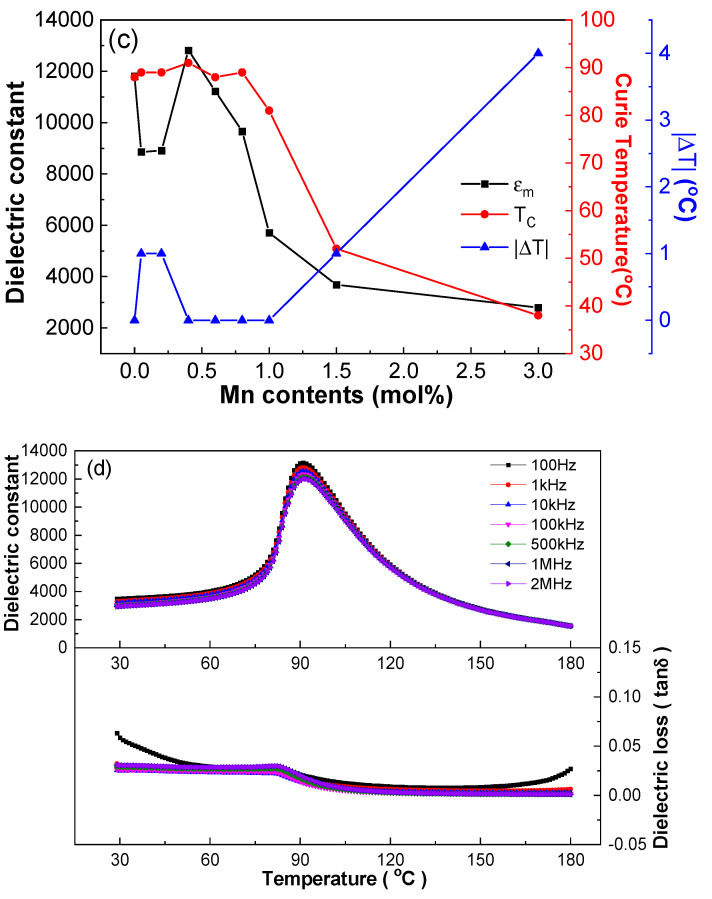
(**a**) Dielectric properties–temperature relationship of BCDTZT-0.4 mol% MnO_2_ ceramics under different sintering temperatures at 1 kHz; (**b**) dielectric properties–temperature relationship of BCDTZT-x mol% MnO_2_ ceramics sintered at 1425 °C at 1 kHz; (**c**) relationship between ε_m_, T_C,_ and |ΔT|_100 Hz-2 MHz_ with MnO_2_ doping amount; (**d**) effect of frequency on dielectric properties–temperature relationship of BCDTZT-0.4 mol% MnO_2_ ceramics sintered at 1425 °C.

Dielectric performance is one of the key factors characterizing the electrical properties of ceramics. Figure 5a shows the dielectric temperature spectra of BCDTZT-0.4 mol% MnO_2_ ceramics sintered at different temperatures from room temperature to 180 °C at 1 kHz. All ceramics exhibited a sole dielectric peak near the Curie temperature (T_C_) of 91 °C, corresponding to a ferroelectric–paraelectric phase transition from the tetragonal phase to the cubic phase [17,22,25]. The sintering temperature affected the dielectric properties of ceramics significantly, in which the value of the maximum dielectric constant (ε_m_) continued to increase while the dielectric loss (tanδ) decreased as the sintering temperature increased. At a sintering temperature of 1450 °C, the ε_m_ value was 14,575 and the dielectric loss was less than 0.025, which was mainly related to its highest bulk density. The high ε_m_ and low tanδ make these ceramics advantageous for obtaining excellent electrical properties [16,17,19].

Figure 5b shows the temperature-dependent dielectric performance of the BCDTZT-x mol% MnO_2_ ceramics sintered at 1425 °C, tested at 1 kHz to investigate the effect of the MnO_2_ doping content on the dielectric properties. It can be seen that the dielectric peak gradually widened with the increase in the MnO_2_ doping content, and the T_C_ temperature gradually shifted towards room temperature, indicating that the relaxation degree of the ceramics was enhanced. This phenomenon was due to the introduction of Mn^4+^ and Mn^2+^ occupying the B-site, causing the distortion of the perovskite structure and the generation of oxygen vacancies [16,17,18,22].

The changes in ε_m_ and T_C_ with the MnO_2_ doping amount in the BCDTZT-x mol% MnO_2_ (x = 0, 0.05, 0.2, 0.4, 0.6, 0.8, 1, 1.5, 3) ceramics are shown in Figure 5c. The ε_m_ value showed a transverse “S”-shape change trend, i.e., decreasing first, then increasing, then decreasing again. The T_C_ temperature stabilized at around 90 °C within x = 0–0.8. When x = 0.4, the ε_m_ value reached the maximum value of 12,817 compared to 11,808 for the undoped ceramic, indicating that an appropriate MnO_2_ doping amount can improve the dielectric properties of BCZT-based ceramics while also stabilizing the T_C_ temperature.

Figure S3 and Figure 5d show the influence of frequency on the dielectric constant and corresponding tanδ of the BCDTZT-x mol% MnO_2_ ceramics in the range of 100 Hz–2 MHz. All samples had broad dielectric peaks and exhibited significant frequency dispersion around T_C_. As shown in Figure 5c, the relaxation degree could be characterized by the |ΔT|_100 Hz-2 MHz_ calculation, and the value was 4 °C at x = 3 mol%, showing obvious diffusive phase transition characteristics. Moreover, from Figure S3f–g, it can be seen that the abnormal dielectric loss change existed in samples at x = 1.5 and 3. When the test temperature was increased from 180 °C to 350 °C, the dielectric loss increased sharply in the high temperature zone, especially at low frequencies. Such abnormal increases could be attributed to the formation of oxygen vacancy defects due to the introduction of Mn^2+^ according to Equations (2)–(4). At high temperatures, oxygen vacancies are excited to generate a large number of hole carriers in ceramics, which induce transition relaxation, increase conductivity, and enlarge dielectric loss [22,36].

The relaxation performance of ferroelectric ceramics with diffusive phase transition can generally be characterized by the exponential law shown below:

(5) $$\frac{1}{\varepsilon }-\frac{1}{\varepsilon_{m}}=\frac{{\left(T-T_{m}\right)}^{\gamma }}{C^{\prime }}$$

**Figure 6:**
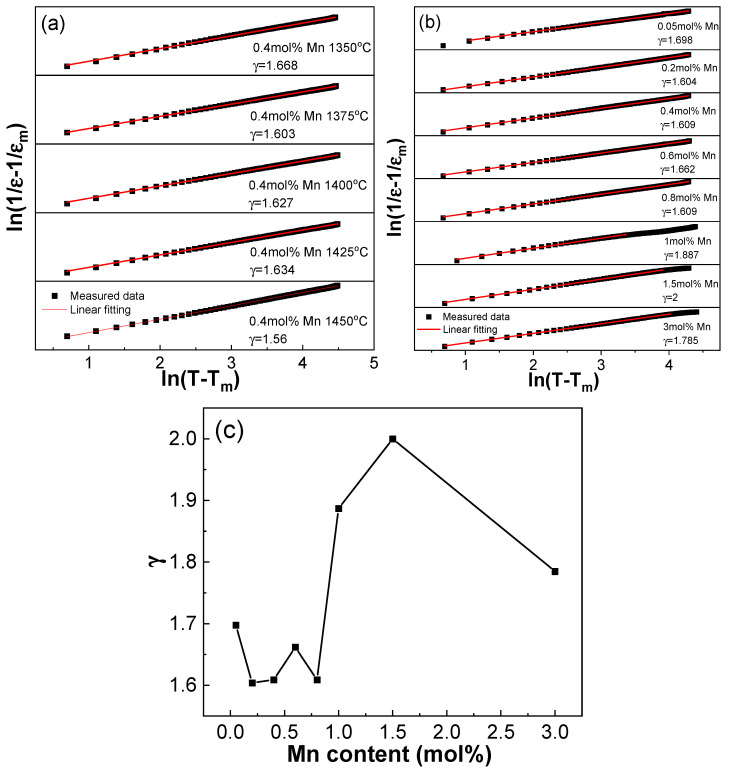
(**a**) Exponential law fitting of BCDTZT-0.4 mol% MnO_2_ ceramics sintered at different temperatures at 1 kHz; (**b**) exponential law fitting of BCTZT-x mol% MnO_2_ ceramics sintered at 1425 °C at 1 kHz; (**c**) relationship between dispersion index γ and MnO_2_ addition amount.

Here, γ represents the dispersion index [19,22,31]. When γ = 1, the material is considered a normal ferroelectric, and when γ = 2, it is considered a relaxor ferroelectric. Figure 6a shows the change in the dispersion factor γ for the BCDTZT-0.4 mol% MnO_2_ ceramics prepared at different sintering temperatures. With increases in the sintering temperature, the γ value did not change significantly, and the value remained stable at around 1.6, indicating that the sintering temperature exerted no significant effect on the relaxation characteristic of the ceramics. As shown in Figure 6b,c, the γ value of BCDTZT-x mol% MnO_2_ varied within the range of 1.6 to 2, presenting certain dispersion characteristics and approaching typical relaxor ferroelectric [36]. When x = 1.5, the γ value was γ = 2, indicating that the BCDTZT-1.5 mol% MnO_2_ ceramic was an ideal relaxor ferroelectric. The relaxation behavior of ceramics could be attributed to the cation disorder caused by the introduction of MnO_2_ at the B-site combined with the coexistence of multiple ions, which disrupted the long-range ordering of ferroelectric materials [17,19,22].

**Figure 7:**
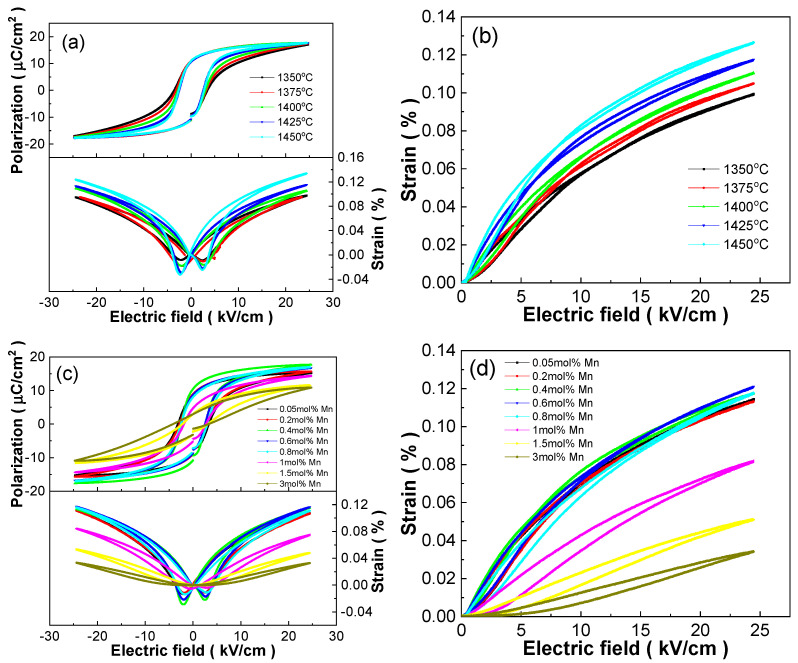
BCDTZT-0.4 mol% MnO_2_ ceramics sintered at different temperatures: (**a**) bipolar P-E loops and S-E curves; (**b**) unipolar S-E curves; BCDTZT-x mol% MnO_2_ ceramics sintered at 1425 °C: (**c**) bipolar P-E loops and S-E curves; (**d**) unipolar S-E curves.

Figure 7 shows the bipolar hysteresis loops (P-E) and electro-induced strain curves (S-E) of BCDTZT-0.4 mol% MnO_2_ ceramics sintered at different temperatures and BCDTZT-x mol% MnO_2_ ceramics sintered at 1425 °C at room temperature, as well as the corresponding unipolar S-E curves. Under the testing conditions of 25 kV/cm and 1 Hz, the P-E loops of all ceramics approached saturation, and the S-E curves showed a typical butterfly shape, indicating that the samples had achieved saturated polarization under these testing conditions. The effect of the sintering temperature on the ferroelectric and strain properties of BCDTZT-0.4 mol% MnO_2_ ceramics can be derived from Figure 7a,b, and the specific changes are given in Table 2. The variation in P-E curves was characterized by maximum polarization (P_max_), remnant polarization (P_r_), and the coercive field (E_c_) while the variation in S-E curves was characterized by maximum strain (S_max_), strain hysteresis (Hys), and the inverse piezoelectric coefficient (d_33_*) [15,22]. From Table 2, it can be seen that the P_max_ value continued to increase with the increase in the sintering temperature while the E_c_ value showed a decreasing trend. At the sintering temperature of 1450 °C, P_max_, P_r_, and E_c_ all reached their respective extreme values. The large P_max_ and P_r_ values, as well as the low E_c_ value, indicate that the ceramics were prone to polarization and were expected to achieve excellent piezoelectric properties. At the same time, the strain performance also improved with the increase in the sintering temperature. At 1450 °C, the ceramic also exhibited excellent strain performance, in which S_max_ was 0.127%, d_33_* was 517.2 pm/V, and Hys was extremely low at 1.73%. The changes in the ferroelectric and strain properties of the BCDTZT-0.4 mol% MnO_2_ ceramics fabricated at different sintering temperatures could also reflect their dependence on grain size [37,38].

The effect of the MnO_2_ doping amount on the ferroelectric and strain properties of ceramics can be determined from Figure 7c,d and Table 3 at the sintering temperature of 1425 °C to investigate the possibility of reducing sintering temperature while optimizing ceramic properties in BCZT-based ceramics by MnO_2_ doping. It can be observed that as the MnO_2_ doping amount increased, both the P_max_ and P_r_ values first increased and then decreased. Meanwhile, the E_c_ value showed an opposite change trend, with a maximum P_max_ value of 17.68 µC/cm^2^ and P_r_ value of 10.64 µC/cm^2^ and a smaller E_c_ value of 2.59 kV/cm at x = 0.4. Large P_r_ and low E_c_ values can be applied to high-density low-electric-field sensors and actuators [3,5,6]. When the MnO_2_ doping content was greater than 0.8, the abnormal decrease in the P_r_ may have been due to segregation at the grain boundary and suppressing domain wall motion caused by excessive MnO_2_ doping amounts [17,20,21], which was consistent with the SEM results. The strain performance presented a similar change trend, with a maximum S_max_ value of 0.121% at x = 0.6. When x < 1, all samples exhibited extremely low Hys values, with values lower than 3%. The x = 0.4 sample had a high S_max_ of 0.118% and a large d_33_* of 480.9 pm/V, presenting the best comprehensive performance sintered at 1425 °C, and could achieve broad application prospects in the field of the electronic components of low-electric-field sensors and actuators [2,3,5,6].

**Figure 8:**
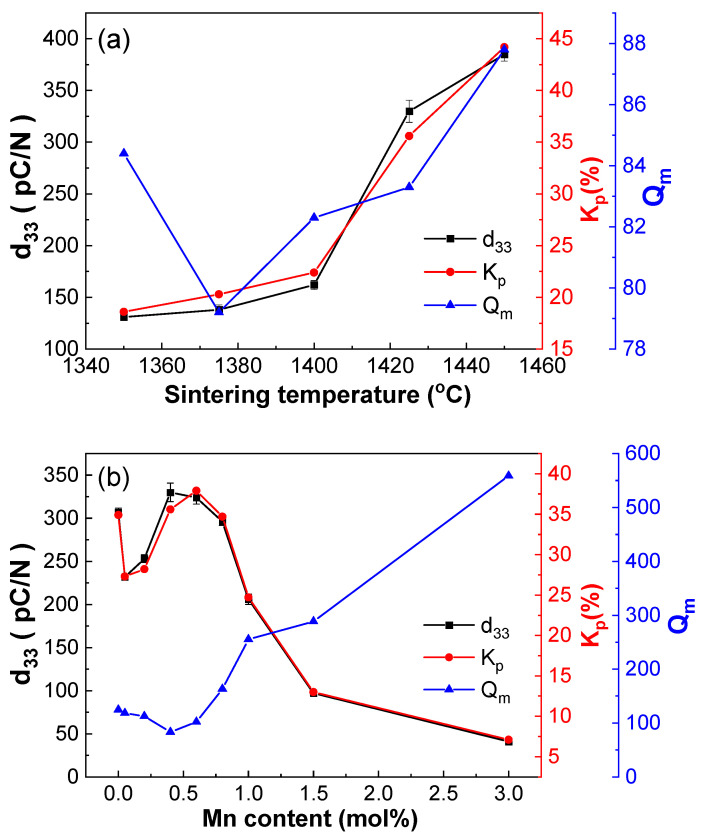
(**a**) Effects of sintering temperature on d_33_, K_p_, and Q_m_ of BCDTZT-0.4 mol% MnO_2_ ceramics; (**b**) effects of MnO_2_ doping amount on d_33_, K_p_, and Q_m_ of BCDTZT-x mol% MnO_2_ ceramics sintered at 1425 °C.

Using the DCP method, all ceramics were polarized at 25 kV/cm for 3 min, then the piezoelectric properties were tested. As shown in Figure 8a, the piezoelectric constant (d_33_), electromechanical coupling coefficient (K_p_), and mechanical quality factor (Q_m_) of BCDTZT-0.4 mol% MnO_2_ ceramics varied with the sintering temperature. d_33_ and K_p_ showed the same change trend as P_r_, reaching their maximum values (d_33_ = 385 pC/N and K_p_ = 0.442) at the sintering temperature of 1450 °C, indicating a direct relationship between the piezoelectric and ferroelectric properties. Within the utilized sintering temperature range, the Q_m_ value fluctuated between 79.2 and 87.8, indicating that the BCDTZT-0.4 mol% MnO_2_ ceramics were soft piezoelectric materials [37].

Figure 8b shows the d_33_, K_p,_ and Q_m_ values of BCDTZT-x mol% MnO_2_ ceramics with different MnO_2_ doping contents sintered at 1425 °C. When the MnO_2_ doping amount was 0.4 mol%, compared with the undoped BCDTZT sample sintered at 1515 °C, the d_33_ value of this ceramic increased from 307 pC/N to 330 pC/N. This indicates that the introduction of MnO_2_ can increase ceramic density while reducing the sintering temperature, which can reduce energy loss and improve the piezoelectric properties of ceramics. According to Equation (6),

(6) $$d_{33}=2Q_{11}\cdot P_{r}\cdot \varepsilon_{33}.$$

Here, Q_11_ is the electro-strictive coefficient and ε_33_ is the dielectric constant [22,39]. It can be seen that the piezoelectric constant is directly proportional to the P_r_ and dielectric constant, which is consistent with the previous results shown in Figure 5c. The Q_m_ value of ceramics tends to increase with an increase in the MnO_2_ doping amount. At x = 3, Q_m_ increases to 558.8, which represents a typical hard piezoelectric material, and presents a favorable candidate material for high-power piezoelectric application [6]. The abnormal increase in Q_m_ in the study was due to the acceptor doping characteristic caused by introducing MnO_2_, which forms defect dipoles and enables ceramics to achieve extremely high Q_m_ values [6,9].

**Figure 9:**
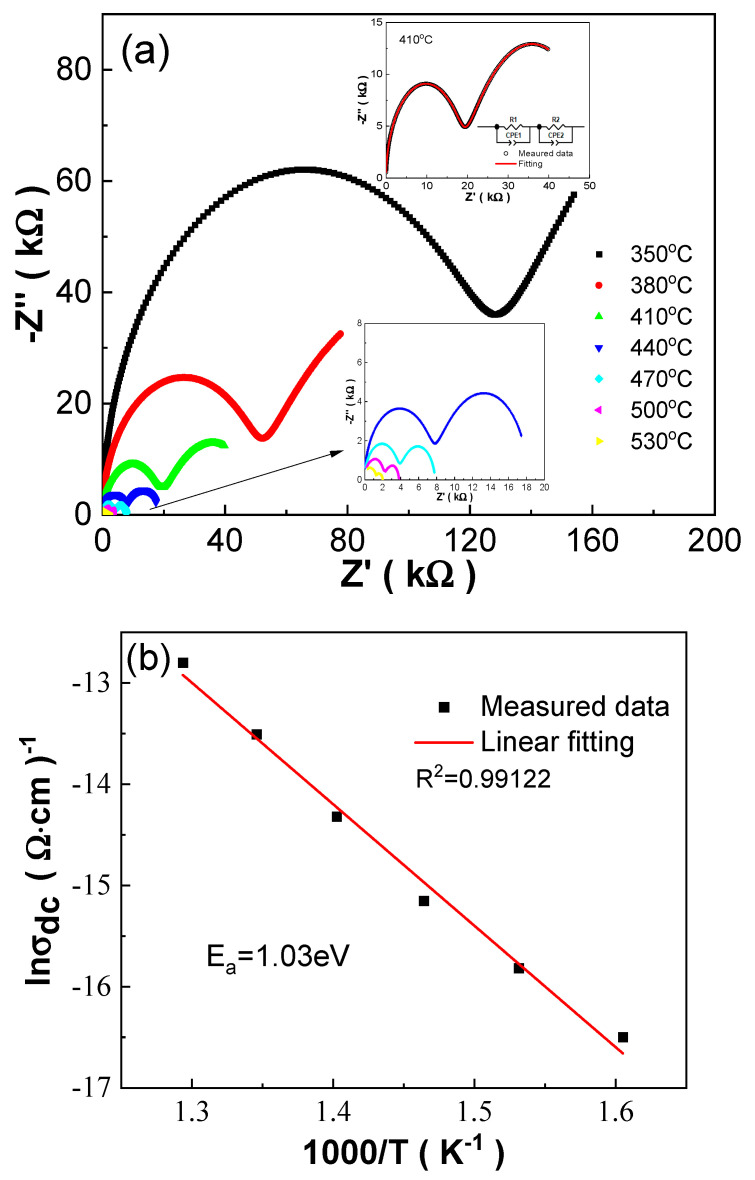

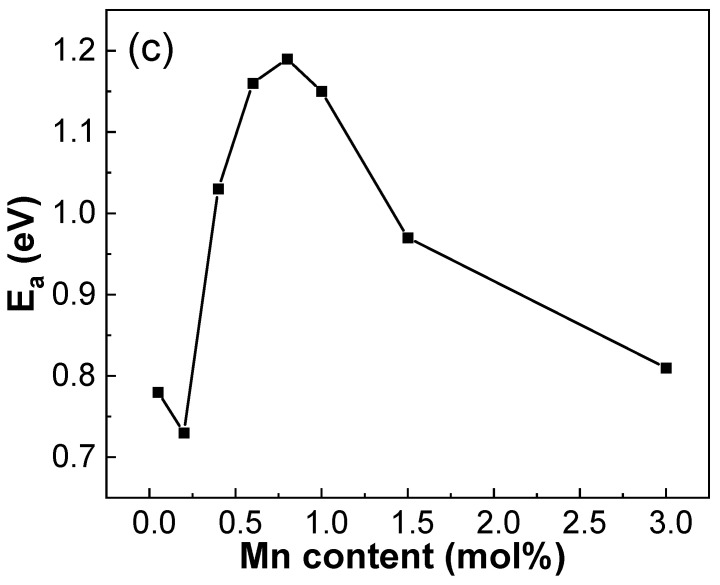
(**a**) Complex impedance spectra of BCDTZT-0.4 mol% MnO_2_ ceramic sintered at 1425 °C. Insets showing a magnified image of high-temperature area and the equivalent circuit fitting curve at 410 °C; (**b**) linear fitting of conductivity–T curve of BCDTZT-0.4 mol% MnO_2_ ceramic using Arrhenius formula; (**c**) variation in E_a_ with MnO_2_ doping amount in BCDTZT-x mol% MnO_2_ ceramics sintered at 1425 °C.

In order to understand the conduction mechanism of BCDTZT-x mol% MnO_2_ ceramics, the relationship between the real part Z′ and imaginary part Z″ of the ceramic impedance was measured in the range of 350–530 °C, and the results are shown in Figure S4 and Figure 9a. It can be seen that at x = 0.05–0.6, there were two clear Cole–Cole semicircles in the high-temperature magnified complex impedance spectra, indicating the existence of two conduction mechanisms, namely grain response and grain boundary response [31,40,41,42]. As the MnO_2_ doping amount continued to increase, the complex impedance spectrum evolved into a Cole–Cole semicircle corresponding to the grain response. The samples with the same sintering temperature exhibited different electrical resistivity values under the same testing conditions, which was due to the variations in density, grain size, and point defects of the ceramics caused by different MnO_2_ doping contents. As the test temperature increased, the radii of all semicircles decreased, indicating a decrease in resistance and reflecting the negative temperature coefficient resistance characteristic of the ceramics [43]. Meanwhile, the Zview software (https://www.ameteksi.com/) was used to perform equivalent circuit fitting on the complex impedance spectra of BCDTZT-x mol% MnO_2_ ceramics at a test temperature of 410 °C, as shown in the illustrations. Among them, R1 represents the equivalent resistance of the grain, R2 represents the equivalent resistance of the grain boundary, C1 represents the capacitance of the grain, and CPE represents the constant phase element. The fitting curves were basically consistent with the test results, indicating the high credibility of equivalent circuit fitting.

According to the Arrhenius formula,

(7) $$\sigma =\sigma_{0}exp(-\frac{E_{a}}{kT})$$

Here, σ is conductivity, σ_0_ is a constant, K is the Boltzmann constant, and T is temperature; the activation energy (E_a_) can be calculated and the conductivity mechanism of the ceramics will be disclosed [41,42].

Figure S5 and Figure 9b show the linear fitting results of the conductivity–T relationship for BCDTZT-x mol% MnO_2_ ceramics, and E_a_ was derived based on the linear fitting. The fitting reliability factor R^2^ of all ceramics was close to 1, and the conductivity activation energy E_a_ was in the range of 0.73 eV–1.19 eV, which was close to the activation energy E_a_~1 eV caused by oxygen vacancy conduction [41,42]. This proves that the conductivity of all ceramics at high temperatures was controlled by oxygen vacancies, which related to the defect reactions (Equations (3) and (4)) caused by the introduction of MnO_2_ as discussed before [22,44]. As shown in Figure 9c, with the increase in the MnO_2_ doping amount, the E_a_ value showed an overall change trend of first increasing and then decreasing, reaching a maximum value of E_a_ = 1.19 eV at x = 0.8.

## 4. Conclusions

The effects of introducing MnO_2_ as a sintering aid on structure and electrical properties of the [(Ba_0.85_Ca_0.15_)_0.999_(Dy_0.5_Tb_0.5_)_0.001_](Zr_0.1_Ti_0.9_)O_3_ ceramics were studied systematically. The results showed that MnO_2_ doping not only reduces the sintering temperature from 1515 °C to 1425 °C but also optimizes the performance of ceramics. The XRD results demonstrated the presence of multiphase coexistence in the ceramics, with x = 0.05~0.8 mol% samples locating at the MPB. The grain size of BCDTZT-x mol% MnO_2_ ceramics decreased while their density increased with a maximum value of 5.38 g/cm^3^ at x = 0.8 mol% and the relative density reached 94.39%. The x = 0.4 mol% sample sintered at 1450 °C had the largest grain size of 11.62 μm. When the MnO_2_ content increased from 0.05 mol% to 1.5 mol%, the dispersion index increased from 1.698 to 2, more approaching typical relaxor ferroelectrics. The 1425 °C sintered x = 0.4 mol% sample was better than the undoped ceramic, in which the optimal performance was ε_m_ = 12,817, d_33_ = 330 pC/N, S_max_ = 0.118%, Hys = 2.66%, and the Curie temperature was maintained at 91 °C. Due to the increase in MnO_2_ content, acceptor doping led to a sharp increase in the Q_m_ value to 558.8 at x = 3 mol%, indicating that moderate MnO_2_ doping can reduce oxygen vacancies. The largest piezoelectric performance with d_33_ = 385 pC/N was obtained in the x = 0.4 mol% sample sintered at 1450 °C. The calculated activation energy E_a_ ranged from 0.73 eV to 1.19 eV, demonstrating that the high-temperature conductivity of ceramics was controlled by oxygen vacancies. This research has shown that the BCDTZT-0.4 mol% MnO_2_ ceramic is a strong candidate material for lead-free piezoelectric ceramics.

## Figures and Tables

**Table 1:** Specific lattice parameters obtained by Rietveld refinement of BCDTZT-x mol% MnO_2_ ceramics.

Sample	Space Group	a (Å)	b (Å)	c (Å)	Phase Fraction (%)	R_wp_ (%)	χ^2^ (%)
x = 0.05	Amm2	3.9962	5.6772	5.7035	68.5	7.55	2.452
	R3m	4.0149	4.0149	4.0149	13.4		
	P4mm	3.9991	3.9991	4.0174	18.1		
x = 0.2	Amm2	3.9908	5.6906	5.7291	50.9	9.2	3.654
	R3m	4.0012	4.0012	4.0012	7.5		
	P4mm	3.9994	3.9994	4.0152	41.6		
x = 0.4	Amm2	3.9957	5.6984	5.6742	41.7	9.28	3.502
	R3m	4.0197	4.0197	4.0197	14.1		
	P4mm	3.9992	3.9992	4.0174	44.2		
x = 0.6	Amm2	3.9967	5.6848	5.6712	45.1	9.74	3.784
	R3m	4.0141	4.0141	4.0141	30.5		
	P4mm	3.9952	3.9952	4.0297	24.4		
x = 0.8	Amm2	3.9834	5.7211	5.6771	54.2	9.99	4.643
	R3m	4.0111	4.0111	4.0111	34.2		
	P4mm	3.9953	3.9953	4.0203	11.6		
x = 1	Amm2	3.9899	5.7178	5.6792	49.8	9.76	4.212
	R3m	4.019	4.019	4.019	8		
	P4mm	3.9911	3.9911	4.0476	17.6		
	Pm3m	4.0093	4.0093	4.0093	24.6		
x = 1.5	Amm2	3.9949	5.6664	5.721	65.4	9.98	4.357
	R3m	4.0051	4.0051	4.0051	12.8		
	P4mm	3.9837	3.9837	4.0367	15.1		
	Pm3m	4.0129	4.0129	4.0129	6.7		
x = 3	Amm2	3.988	5.7029	5.7409	68.1	9.99	4.135
	R3m	4.0051	4.0051	4.0051	9.2		
	P4mm	3.9957	3.9957	4.0378	1		
	Pm3m	4.0145	4.0145	4.0145	21.7		

**Table 2:** Ferroelectric properties of BCDTZT-0.4 mol% MnO_2_ ceramics sintered at 1350 °C~1450 °C.

Sintering Temperature	P_max_ (µC/cm^2^)	P_r_ (µC/cm^2^)	E_c_ (kV/cm)	S_max_ (%)	Hys (%)	d_33_* (pm/V)
1350 °C	17.09	10.87	3.58	0.099	0.33	406.6
1375 °C	17.42	11.00	3.19	0.105	1.90	429.5
1400 °C	17.57	10.78	2.80	0.110	0.30	451.8
1425 °C	17.68	10.64	2.59	0.118	2.66	480.9
1450 °C	17.7	11.37	2.44	0.127	1.73	517.2

**Table 3:** Ferroelectric properties of BCDTZT-x mol% MnO_2_ ceramics sintered at 1425 °C.

Sample	P_max_ (µC/cm^2^)	P_r_ (µC/cm^2^)	E_c_ (kV/cm)	S_max_ (%)	Hys (%)	d_33_* (pm/V)
x = 0.05	15.27	8.92	3.18	0.115	1.07	468.1
x = 0.2	15.71	8.95	2.83	0.113	1.11	462.5
x = 0.6	16.79	8.64	2.45	0.121	0.97	495.1
x = 0.8	17.05	8.02	2.51	0.118	2.43	481.1
x = 1	14.39	4.82	1.91	0.082	8.06	335.0
x = 1.5	11.61	2.99	2.20	0.051	11.40	208.8
x = 3	10.92	3.00	4.22	0.034	15.40	140.1

## Data Availability

All data that support the findings of this study are included within the article and Supplementary Materials or available from the corresponding author upon request.

## Supplementary Materials

The following supporting information can be downloaded at: https://www.mdpi.com/article/10.3390/ma18081888/s1, Figure S1: XRD Rietveld refinement results of BCDTZT-x mol% MnO_2_ ceramics sintered at 1425 °C; Figure S2: SEM microstructure of BCDTZT-0.4 mol% MnO_2_ ceramics at different sintering temperatures; Figure S3: Effect of frequency on dielectric properties-temperature relationship of BCDTZT-x mol% MnO_2_ ceramics sintered at 1425 °C; Figure S4: Complex impedance spectra of BCDTZT-x mol% MnO_2_ ceramics sintered at 1425 °C; Figure S5: Linear fitting of conductivity-T curves of BCDTZT-x mol% MnO_2_ ceramics sintered at 1425 °C using Arrhenius formula.

## References

[1] Yan F., Qian J., Wang S., Zhai J. (2024). Progress and outlook on lead-free ceramics for energy storage applications. Nano Energy.

[2] Sahoo B., Thejas T., Politova E., Panda P. (2021). Effect of dopants on electrical properties of BCT-BZT lead free piezo ceramics: A review. Ferroelectrics.

[3] Panda P., Sahoo B., Thejas T., Krishna M. (2022). High d_33_ lead-free piezoceramics: A Review. J. Electron. Mater..

[4] Li Z., Yu J., Hao S., Janolin P.-E. (2022). Enhancing properties of lead-free ferroelectric BaTiO_3_ through doping. J. Eur. Ceram. Soc..

[5] Verma R., Chauhan A., Batoo K., Jasrotia R., Sharma A., Kumar R., Hadi M., Raslan E., Labis J., Imran A. (2021). Review—Modulation of Dielectric, Ferroelectric, and Piezoelectric Properties of Lead-Free BCZT Ceramics by Doping. ECS J. Solid State Sci. Technol..

[6] Nguyen T.N., Thong H.-C., Zhu Z.-X., Nie J.-K., Liu Y.-X., Xu Z., Soon P.-S., Gong W., Wang K. (2021). Hardening effect in lead-free piezoelectric ceramics. J. Mater. Res..

[7] Liu W., Ren X. (2009). Large piezoelectric effect in Pb-free ceramics. Phys. Rev. Lett..

[8] Coondoo I., Pullar R.C., Miranda G. (2024). Multifunctional lead-free piezoelectric (Ba, Ca)(Zr, Ti)O_3_ compounds: From energy harvesting to electrocaloric cooling and energy storage applications. Mater. Res. Bull..

[9] Buatip N., Munthala D., Janphuang P., Pojprapai S. (2024). Investigation of energy harvesting performance of BCZT piezoelectric ceramics under low frequency. Bull. Mater. Sci..

[10] Thakur N., Gopalan P., Kolte J. (2024). Structural, electrical, and dynamic scaling behavior of Ba_0.85_Ca_0.15_Zr_0.10_Ti_0.90_O_3_ nanoceramics synthesized at low temperature by sonochemical method. Ceram. Int..

[11] Yang P., Zhao L., Shi S., Zheng H., Yu S. (2024). Effects of multiple sintering additives on crystal structure, morphology and tunable mechanisms of BCZT ceramics. J. Mater. Sci..

[12] Kumari S., Kumar A., Kumar V., Aggarwal S., Goyal P.K., Gaur A., Arya A., Kumar A. (2023). Enhanced Curie temperature with a significant reduction in sintering temperature for Cu^2+^/Bi^3+^ co-doped BCZT lead-free ceramics. Mater. Sci. Eng. B.

[13] Jaiban P., Theethuan T., Khumtrong S., Lokakaew S., Watcharapasorn A. (2022). The effects of donor (Nb^5+^) and acceptor (Cu^2+^, Zn^2+^, Mn^2+^, Mg^2+^) doping at B-site on crystal structure, microstructure, and electrical properties of (Ba_0.85_Ca_0.15_)Zr_0.1_Ti_0.9_O_3_ ceramics. J. Alloys Compd..

[14] Ma P., Wang J., He Y., Duan X. (2024). Dielectric and Ferroelectric Performances of Y, Mn Co-doped Barium Calcium Zirconate Titanate Based Lead-Free Piezoceramics. J. Mater. Eng. Perform..

[15] Du J., Qiu L., Yang C., Zheng H., Zhu K., Wang L. (2022). Structure and electrical properties in CuO-modified BCZT lead-free piezoelectric ceramics. J. Electroceram..

[16] Yu Y., Guo W., Zhen Y., Cen Z., Ji A., Wu H., Liang S., Xiong S., Wang X. (2023). Influence of MnO_2_ addition on the dielectric properties of 0.95MgTiO_3_-0.05CaTiO_3_ ceramics sintered in a reducing atmosphere. J. Eur. Ceram. Soc..

[17] Zheng Y., Shi Y., Ren Z., Zhang B., Feng J., Li H., Dang S., Yang F., Shang J., Yin S. (2022). Preparation and electrical properties of Ba_0.85_Ca_0.15_Zr_0.1_Ti_0.9_O_3_ ceramics by the doping of Mn ions. Physica B.

[18] Kar K.S., Chandrasekhar M., Rao L.T., Harshavardhan V., Kumar P. (2024). Effect of MnO_2_ addition on structure, electrical and optical properties of Ba(Fe_0.5_Nb_0.5_)O_3_ ceramics. Process. Appl. Ceram..

[19] Peng W., Li L., Yu S., Yang P., Xu K. (2021). Dielectric properties, microstructure and charge compensation of MnO_2_-doped BaTiO_3_-based ceramics in a reducing atmosphere. Ceram. Int..

[20] Li Z., Xun W., Huang X., Wan Y., Liu Y., Gu S., He W., Yang W., Lin Z., Wang B. (2024). Significant enhancement of ferroelectric performance in lead-free NaNbO_3_ ceramics. Ceram. Int..

[21] Lin J., Qin S., Cui B., Cheng J., Chen J. (2023). Reduced dielectric loss and improved electric thermal stability of BF–PT–BT ceramics by Mn additions. J. Mater. Sci..

[22] Mekonnen M.A., Tadesse M.Z. (2021). Low temperature sintering of (Ba_0.85_Ca_0.15_)(Ti_0.90_Zr_0.10_)O_3_ lead-free piezoceramic with the additive of MnO_2_. J. Electroceram..

[23] Wang R., Cheng Y., Xu S., Zhang G., Zhang H., Li W. (2025). Tb^3+^/Dy^3+^ doped glass ceramics containing Bi_2_Ti_2_O_7_ crystal phases for luminescence and temperature sensing. Ceram. Int..

[24] Ke L., Ren K., Cai X., Zhang Y. (2024). Energy transfer and color tunability in high-thermal-stability Dy^3+^/Tb^3+^ co-doped K_3_YF_6_ transparent oxyfluoride glass ceramics. J. Alloys Compd..

[25] Kim T.W., Lee G., Ichimura M., Koh J.-H. (2024). Enhanced soft piezoelectric properties of Sb_2_O_3_ doped 0.5Ba(Zr_0.2_Ti_0.8_)O_3_-0.5(Ba_0.7_Ca_0.3_)TiO_3_ materials. J. Alloys Compd..

[26] Zhang L., Zhang M., Wang L., Zhou C., Zhang Z., Yao Y., Zhang L., Xue D., Lou X., Ren X. (2014). Phase transitions and the piezoelectricity around morphotropic phase boundary in Ba (Zr_0.2_Ti_0.8_)O_3_-x(Ba_0.7_Ca_0.3_)TiO_3_ lead-free solid solution. Appl. Phys. Lett..

[27] Acosta M., Khakpash N., Someya T., Novak N., Jo W., Nagata H., Rossetti Jr G.A., Rödel J. (2015). Origin of the large piezoelectric activity in (1− x)Ba(Zr_0.2_Ti_0.8_)O_3_-x(Ba_0.7_Ca_0.3_)TiO_3_ ceramics. Phys. Rev. B.

[28] Coondoo I., Panwar N., Krylova S., Krylov A., Alikin D., Jakka S.K., Turygin A., Shur V.Y., Kholkin A.L. (2021). Temperature-dependent Raman spectroscopy, domain morphology and photoluminescence studies in lead-free BCZT ceramic. Ceram. Int..

[29] Dhifallah N., Hentati M.A., Khemakhem H. (2024). Orthorhombic–tetragonal phase coexistence and enhanced piezoelectric properties at room temperature in Zn and Ta modified (Ba_0.95_Ca_0.05_)(Zr_0.05_Ti_0.95_)O_3_ ceramics through the synergistic effect of lattice distortion. RSC Adv..

[30] Fang Y., Shui A., Yu H., Zhong X. (2024). High energy storage performance in SrZrO_3_-modified quaternary relaxor ferroelectric ceramics. Ceram. Int..

[31] Yan G., Sun J., Yan J., Deng T., Fang B., Hao J., Zhang S., Lu X., Zhao X., Ding J. (2023). Pulse energy-storage performance and temperature stability of Bi_2_O_3_-added BaTiO_3_ based ceramics. Ceram. Int..

[32] Kröger F.A., Vink H.J. (1956). Relations between the concentrations of imperfections in crystalline solids. Solid State Phys..

[33] Yu Y., Zheng T., Zhang N., Wu J. (2022). Review of Sintering Aids in Lead-Free (K, Na)NbO₃-Based Ceramics. IEEE Trans. Ultrason. Ferroelectr. Freq. Control.

[34] Yang Z., Yin Z., Wang D., Wang H., Song H., Zhao Z., Zhang G., Qing G., Wu H., Jin H. (2020). Effects of ternary sintering aids and sintering parameters on properties of alumina ceramics based on orthogonal test method. Mater. Chem. Phys..

[35] Ben F., Xu D., Zhou X., Yu T., Wei J., Zhao W. (2025). Crystalline structure and dielectric relaxor behavior of MnO_2_-modified 0.8BaTiO_3_-0.2BiScO_3_ ceramics for energy storage application. Mater. Chem. Phys..

[36] Sun J., Yang Y., Fang B., Zhang S., Lu X., Ding J. (2024). Improving multifunctional performance of Dy-doped (Ba_0.85_Ca_0.15_)(Zr_0.1_Ti_0.9_)O_3_ ceramics via tailoring Dy-doping amount and ceramic processing. Ferroelectrics.

[37] Sun M., Du J., Chen C., Fu P., Li P., Hao J., Yue Z., Li W. (2020). Enhanced piezoelectric properties in M (M = Co or Zn)-doped Ba_0.99_Ca_0.01_Ti_0.98_Zr_0.02_O_3_ ceramics. Ceram. Int..

[38] Amorín H., Venet M., García J.E., Ochoa D.A., Ramos P., López-Sánchez J., Rubio-Zuazo J., Castro A., Algueró M. (2024). Insights into the Early Size Effects of Lead-Free Piezoelectric Ba_0.85_Ca_0.15_Zr_0.1_Ti_0.9_O_3_. Adv. Electron. Mater..

[39] Hao J., Bai W., Li W., Zhai J. (2012). Correlation between the microstructure and electrical properties in high-performance (Ba_0.85_Ca_0.15_)(Zr_0.1_Ti_0.9_)O_3_ lead-free piezoelectric ceramics. J. Am. Ceram. Soc..

[40] Shang M., Ren P., Wan Y., Lu X. (2023). Tailoring Curie temperature and dielectric properties by changing the doping sites of Y ions in (Ba, Ca)(Zr, Ti)O_3_ ceramics. J. Eur. Ceram. Soc..

[41] Wang L., Bai W., Zhao X., Ding Y., Wu S., Zheng P., Li P., Zhai J. (2020). Influences of rare earth site engineering on piezoelectric and electromechanical response of (Ba_0.85_Ca_0.15_)(Zr_0.1_Ti_0.9_)O_3_ lead-free ceramics. J. Mater. Sci. Mater. Electron..

[42] Sun J., Yan G., Fang B., Zhang S., Lu X., Ding J. (2024). Improving energy storage performance of BLLMT ceramic by doping BZT combining with defect engineering and film scraping process. J. Alloys Compd..

[43] Belkhadir S., Moumen S.B., Asbani B., Amjoud M., Mezzane D., Luk’Yanchuk I.A., Choukri E., Hajji L., Gagou Y., El Marssi M. (2019). Impedance spectroscopy analysis of the diffuse phase transition in lead-free (Ba_0.85_Ca_0.15_)(Zr_0.1_Ti_0.9_)O_3_ ceramic elaborated by sol-gel method. Superlattices Microstruct..

[44] Wang X., Huan Y., Zhu Y., Zhang P., Yang W., Li P., Wei T., Li L., Wang X. (2022). Defect engineering of BCZT-based piezoelectric ceramics with high piezoelectric properties. J. Adv. Ceram..

