# Supplementary material for: Reducing Sintering Temperature While Optimizing Electrical Properties of BCZT-Based Lead-Free Ceramics by Adding MnO2 as Sintering Aid
Source: Materials (Basel). 2025 Apr 21;18(8):1888. doi: 10.3390/ma18081888 (PMC12028373; doi:10.3390/ma18081888)
Supplement: Supplementary file 1 [file materials-18-01888-s001.zip › materials-3527814-supplementary.pdf]

Supporting information for

Reducing sintering temperature while optimizing electrical properties of BCZT-based lead-free ceramics by adding MnO_2 as sintering aid

Xinlin Yang[1], Bijun Fang[1,*], Shuai Zhang[1], Xiaolong Lu[1], Jianning Ding[1,2,*]

[1] School of Materials Science and Engineering, Jiangsu Collaborative Innovation Center of Photovoltaic Science and Engineering, State Key Laboratory of Photovoltaic Science and Technology, National Experimental Demonstration Center for Materials Science and Engineering, Changzhou University, Changzhou 213164, China

[2] School of Mechanical Engineering, Yangzhou University, Yangzhou 225127, China

*Corresponding authors.

E-mail addresses: fangbj@cczu.edu.cn (B. Fang), dingjn@cczu.edu.cn (J. Ding)

Tel.: +86 519 86330095; Fax: +86 519 86330095

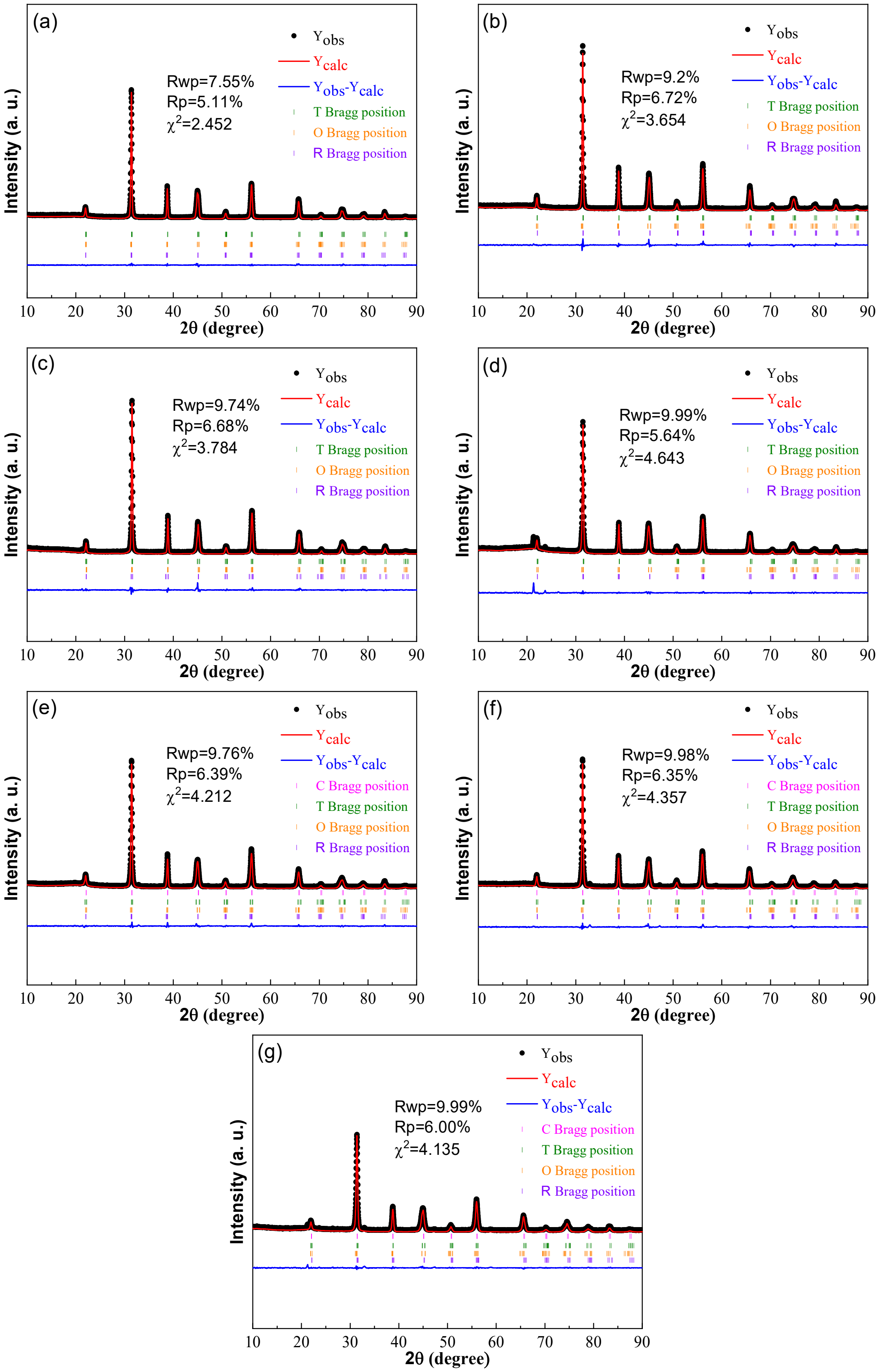

Fig. S1 XRD Rietveld refinement results of BCDTZT-x mol% MnO_2 ceramics sintered at 1425 °C. (a) x=0.05; (b) x=0.2; (c) x=0.6; (d) x=0.8; (e) x=1; (f) x=1.5; (g) x=3.

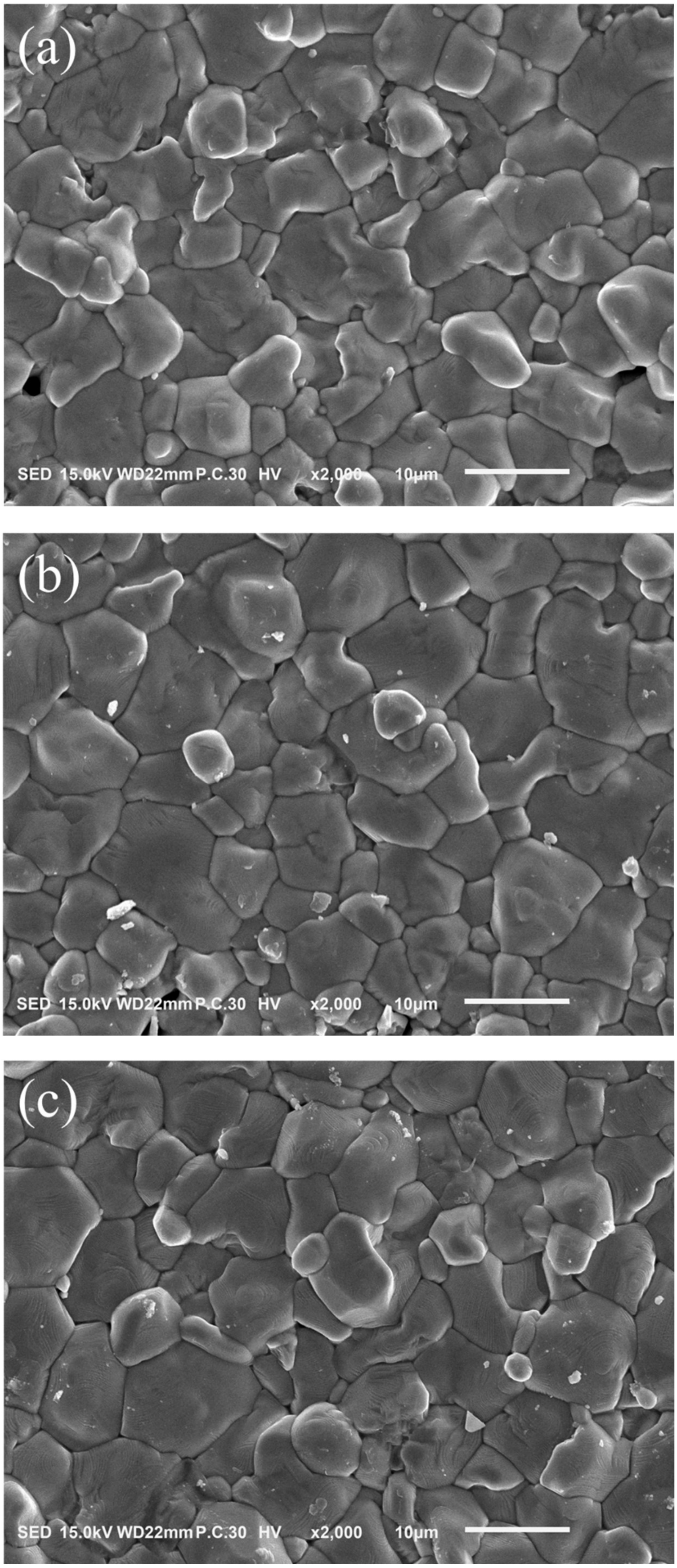
(a)
SED 15.0kV WD22mm P.C.30 HV x2,000 10µm
(b)
SED 15.0kV WD22mm P.C.30 HV x2,000 10µm
(c)
SED 15.0kV WD22mm P.C.30 HV x2,000 10µm

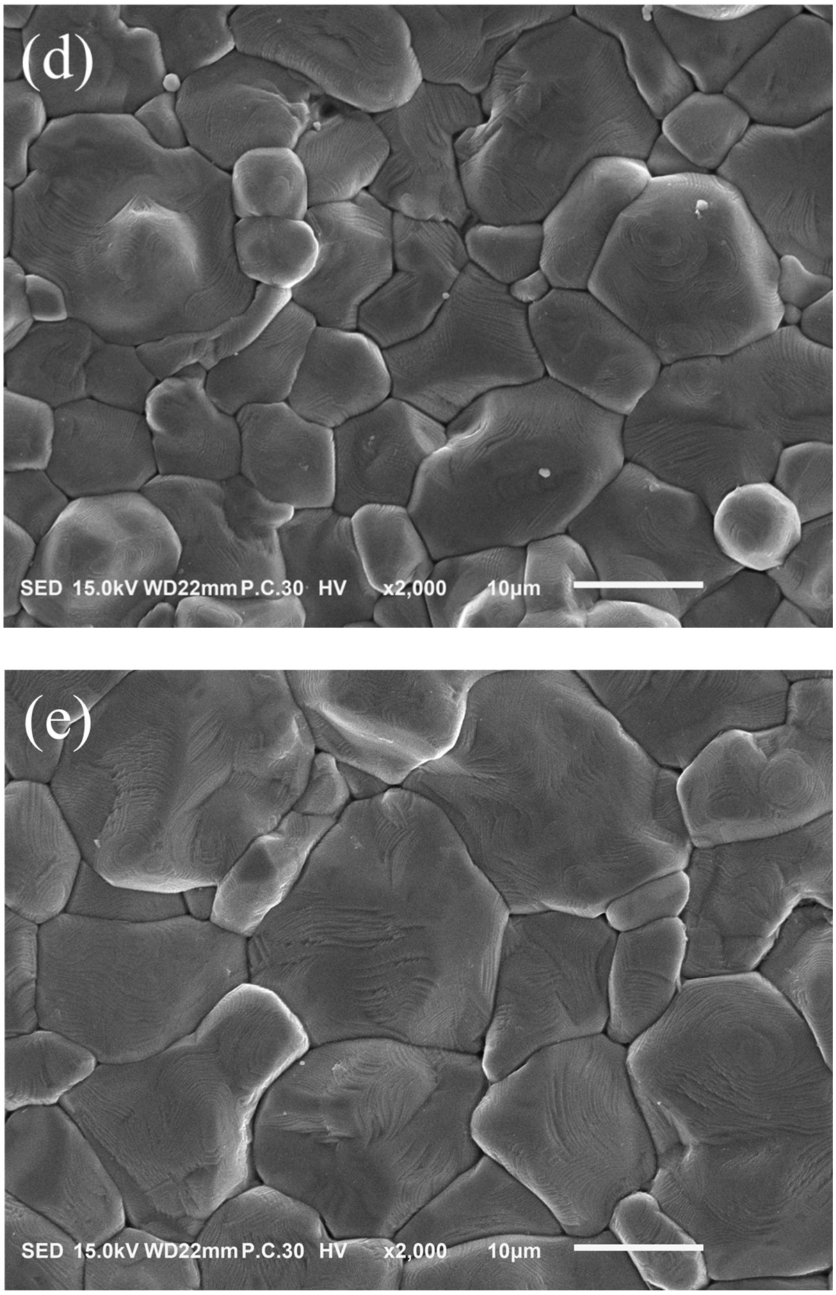

Fig. S2 SEM microstructure of BCDTZT-0.4 mol% MnO_2 ceramics at different sintering temperatures: (a) 1350 °C; (b) 1375 °C; (c) 1400 °C; (d) 1425 °C; (e) 1450 °C.

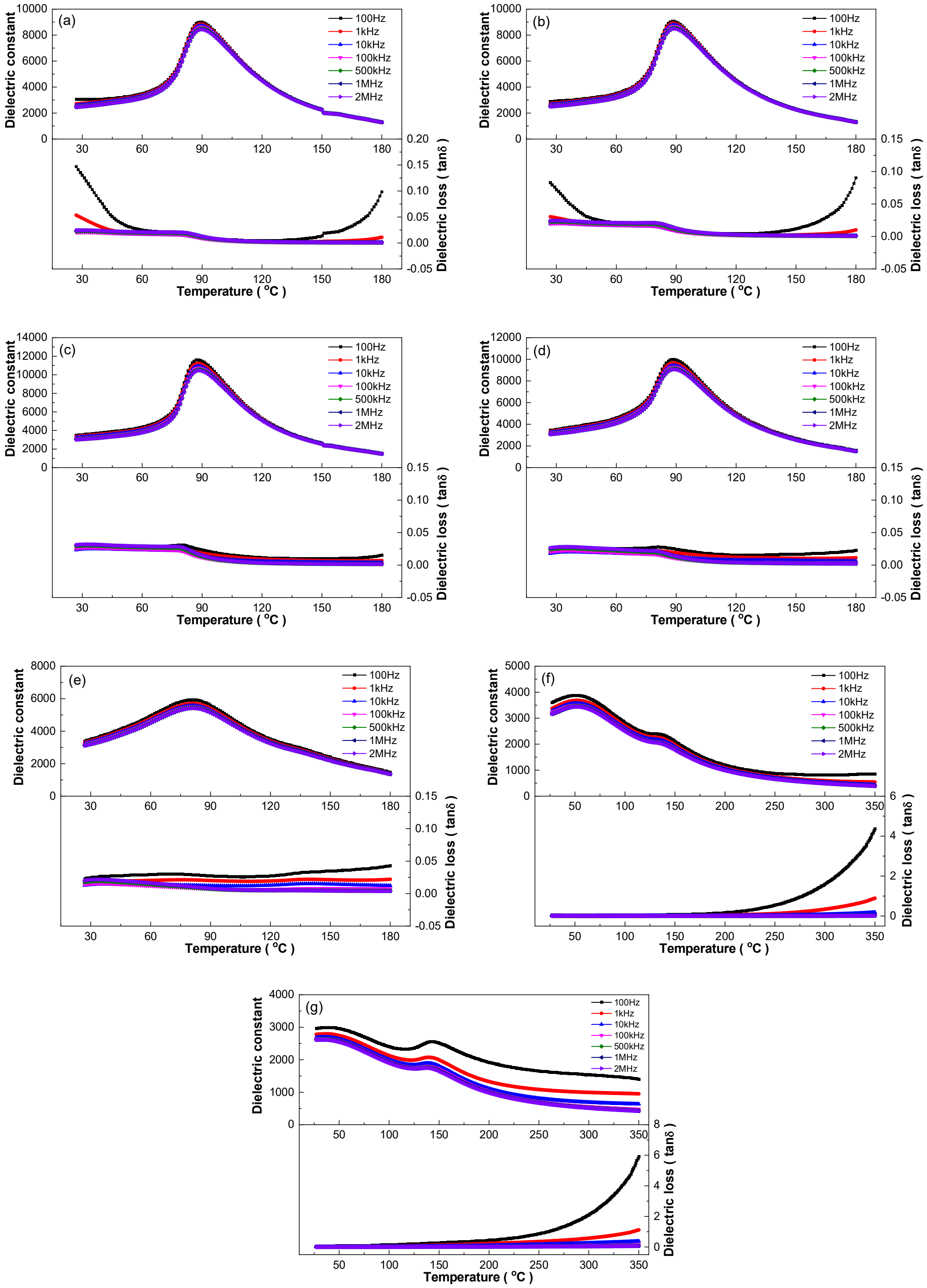

Fig. S3 Effect of frequency on dielectric properties-temperature relationship of BCDTZT-x mol% MnO_2 ceramics sintered at 1425 °C.

(a) x=0.05; (b) x=0.2; (c) x=0.6; (d) x=0.8; (e) x=1; (f) x=1.5; (g) x=3.

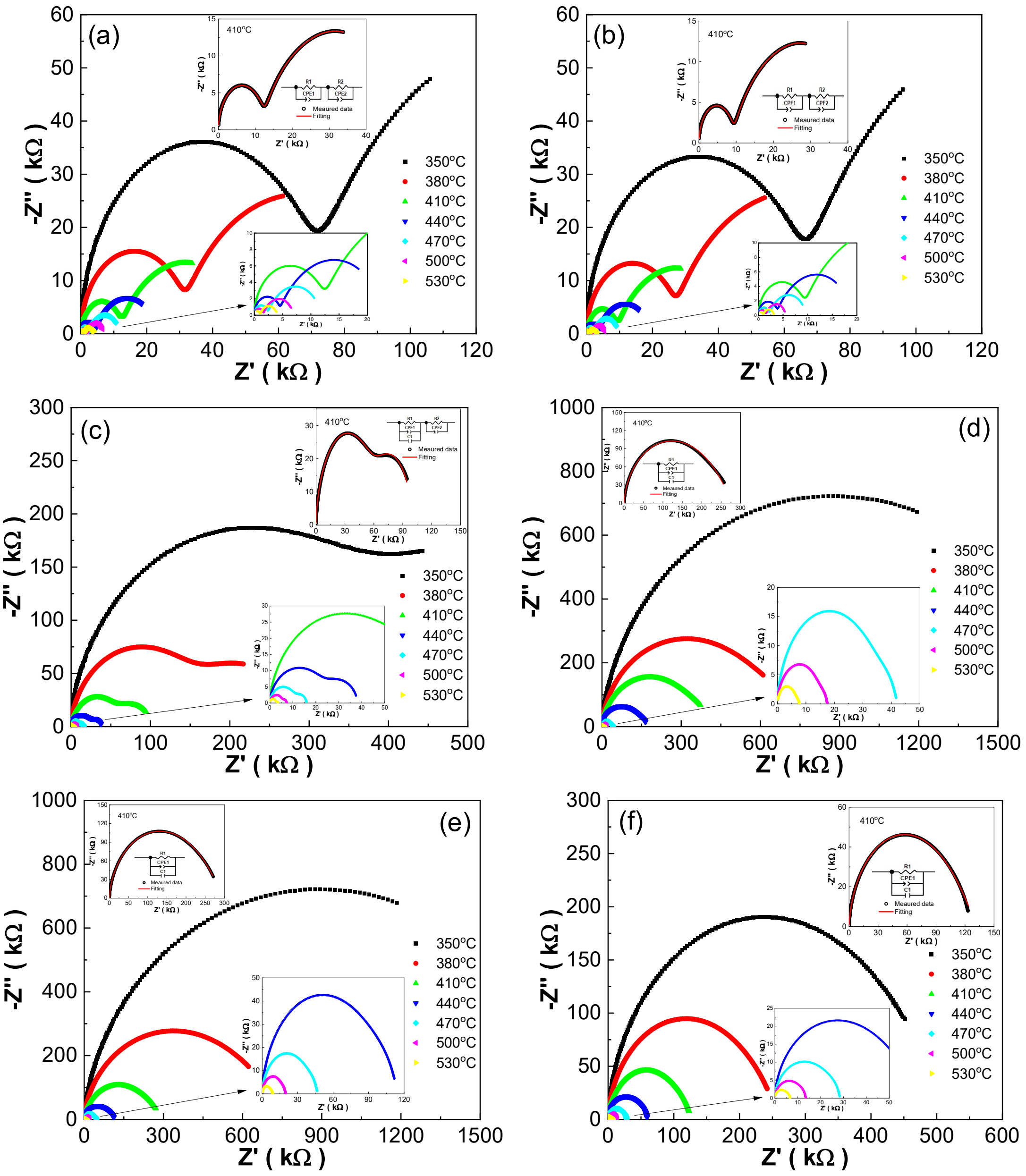

(a)
(b)
(c)
(d)
(e)
(f)
-Z'' (kΩ)
Z' (kΩ)
350°C
380°C
410°C
440°C
470°C
500°C
530°C
410°C
Meaured data
Fitting
R1
R2
CPE1
CPE2
C1

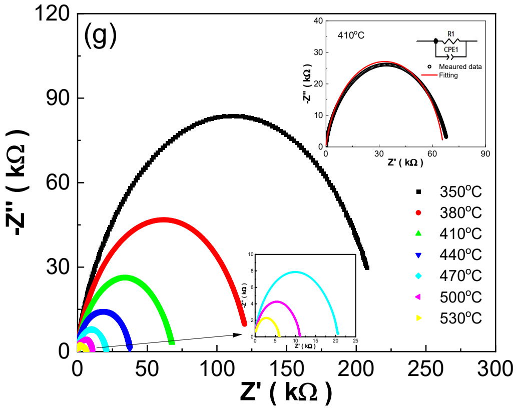

Fig. S4 Complex impedance spectra of BCDTZT-x mol% MnO_2 ceramics sintered at 1425 °C.

(a) x=0.05; (b) x=0.2; (c) x=0.6; (d) x=0.8; (e) x=1; (f) x=1.5; (g) x=3.

Insets showing magnified images of high-temperature area and equivalent circuit fitting curves at 410 °C.

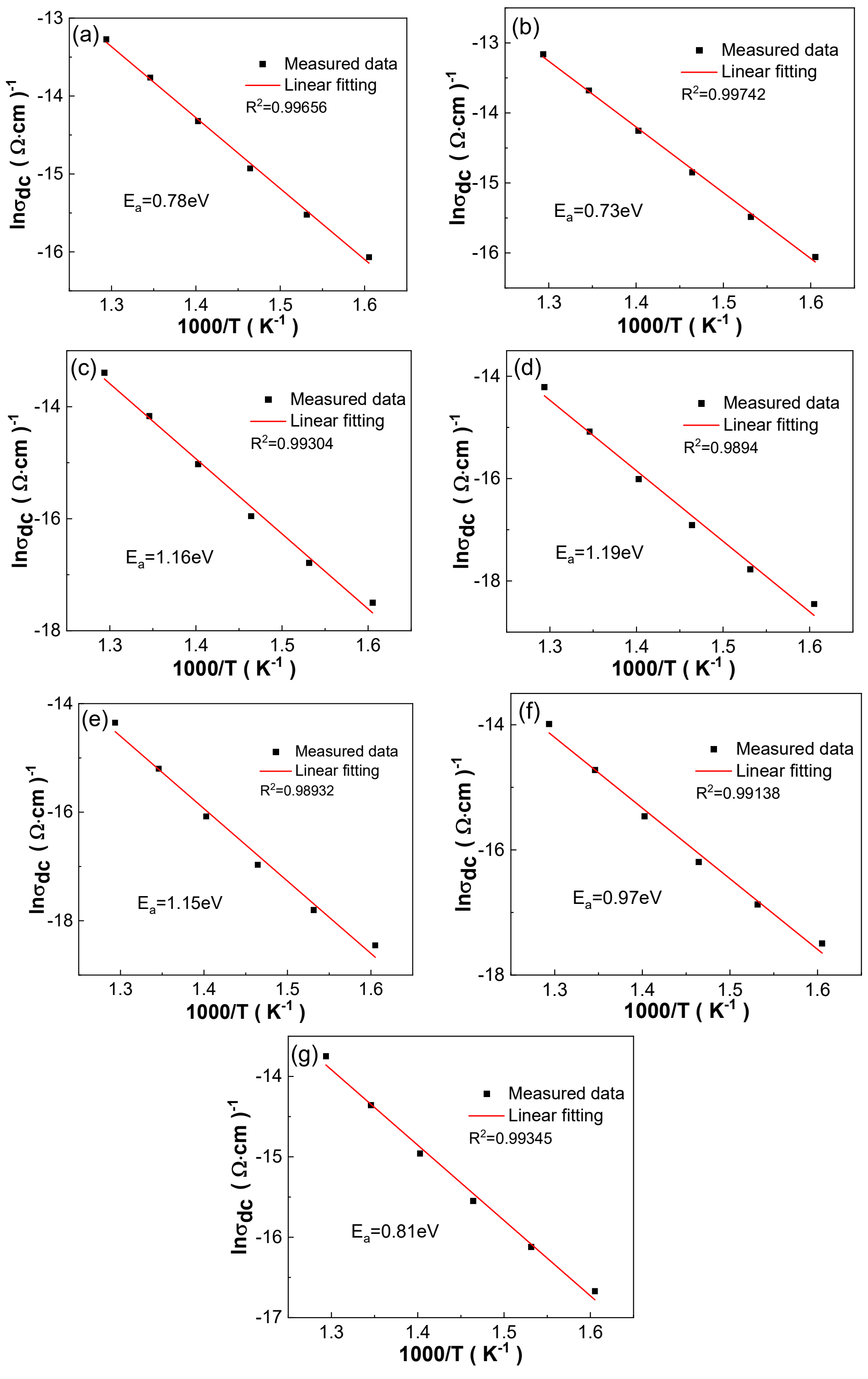

Fig. S5 Linear fitting of conductivity-T curves of BCDTZT-x mol% MnO_2 ceramics sintered at 1425 °C using Arrhenius formula.

(a) x=0.05; (b) x=0.2; (c) x=0.6; (d) x=0.8; (e) x=1; (f) x=1.5; (g) x=3.
